# Computer-Aided Design of Cefuroxime Axetil/Cyclodextrin System with Enhanced Solubility and Antimicrobial Activity

**DOI:** 10.3390/biom10010024

**Published:** 2019-12-23

**Authors:** Mikołaj Mizera, Daria Szymanowska, Anna Stasiłowicz, Dominika Siąkowska, Kornelia Lewandowska, Andrzej Miklaszewski, Tomasz Plech, Ewa Tykarska, Judyta Cielecka-Piontek

**Affiliations:** 1 Department of Pharmacognosy, Faculty of Pharmacy, Poznań University of Medical Sciences, Święcickiego 4, 60-781 Poznań, Poland; mikolajmizera@gmail.com (M.M.); stasilowicz.anna@gmail.com (A.S.); dominika.siakowska@interia.eu (D.S.); 2 Department of Biotechnology and Food Microbiology, Poznan University of Life Sciences, Wojska Polskiego 48, 60-627 Poznan, Poland; darszy@up.poznan.pl; 3 Institute of Molecular Physics, Polish Academy of Science, ul. Smoluchowskiego 17, 60-179 Poznań, Poland; kornelia.lewandowska@ifmpan.poznan.pl; 4 Poznan University of Technology, Institute of Materials Science and Engineering, Pl. M.Sklodowskiej-Curie 5, 60-965 Poznan, Poland; andrzej.miklaszewski@put.poznan.pl; 5 Department of Pharmacology, Medical University of Lublin, Chodźki 4a, 20-093 Lublin, Poland; tomasz.plech@umlub.pl; 6 Department of Chemical Technology of Drugs, Poznan University of Medical Sciences, Grunwaldzka 6, 60-780 Poznan, Poland; etykarsk@ump.edu.pl

**Keywords:** cefuroxime axetil, cyclodextrin, molecular modeling

## Abstract

This study aimed to investigate changes in the solubility and antimicrobial efficacy of cefuroxime axetil (CA) when incorporated into cyclodextrin (CD). While choosing the CD, the validated in silico model was used. A theoretical model based on docking and molecular mechanics/generalized born surface area was validated using a curated dataset of API (active pharmaceutical ingredient)–CD stability constants. The library of commonly used cyclodextrins was virtually screened, indicating CA –hydroxypropyl-βCD (HPβCD) as the most thermodynamically favored system. Solid-state CA–HPβCD system was prepared and characterized by differential scanning calorimetry (DSC), Fourier-transform infrared (FT-IR), and X-ray diffraction (XRPD) methods. The dissolution profiles of the CA and its cyclodextrin system were evaluated. Microbiological activity of the CA–HPβCD inclusion system was studied based on changes in minimal inhibitory concentration (MIC) values and related to ones of the pure CA. The theoretical model was successfully validated, obtaining an average correlation with experimental data R = 0.7. The dissolution study showed significantly improved dissolution profiles of CA–HPβCD compared to CA. HPβCD increases the antimicrobial efficacy of CA up to 4-fold compared to pure CA.

## 1. Introduction

**Figure 1:**
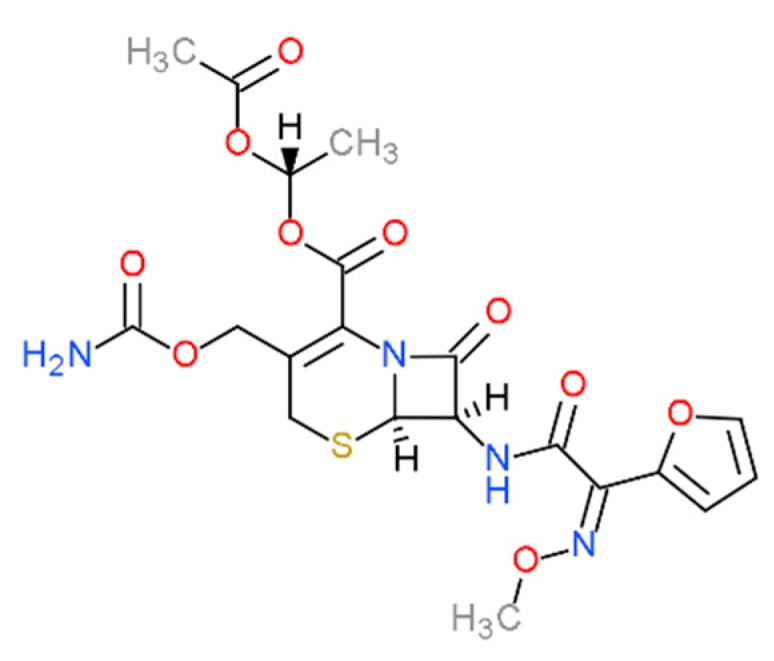
Chemical structure of cefuroxime axetil

Cefuroxime axetil (CA) is an oral cephalosporin prodrug (Figure 1) transformed in vivo to its active form—cefuroxime, the beta-lactam analog. Cefuroxime exhibits a broad spectrum of activity against Gram-negative and Gram-positive bacteria [1]. The main indications include respiratory tract infections, middle ear infections, sinus infections caused by non-β-lactamase-producing strains, and treatment of urinary tract infections also of pediatric patients [2,3].

Esterification of cefuroxime into the prodrug form facilitated its permeation, resulting in increased bioavailability, and enabled the development of oral formulations [4]. However, the change of physicochemical properties of CA relative to its parent molecule (acidic form) entailed poor solubility due to increased lipophilicity [1]. The studies of crystal structures revealed the dependence of the increase in CA solubility on the content of amorphous fraction of formulation [5,6]. Although the amorphous form of CA improves the solubility rate over polymorphs, CA still is classified as BCS (Biopharmaceutics Classification System) Class II.

The solubility of BCS Class II drugs may be enhanced by guest–host inclusion systems with cyclodextrins (CDs). The CDs are a group of glucose polymers, in which cyclic structure creates a lipophilic cavity and enables the formation of inclusion systems. The CDs have proven their usability as solubilization agents in numerous pharmaceutical applications, including systems with β-lactam analogs. In the study of Shah et al., CA was complexed with hydroxypropyl-βCD (HPβCD), resulting in improved solubility [7]. A recent study on the CA–βCD system also showed an increase in the solubility of CA in the system relative to pure CA [8]. The findings related to the high stability of CA–HPβCD are consistent with the increased bioavailability of the system compared to pure CA, reported by Prabhakaran et al. [9].

The ultimate goal of applying systems that increase the bioavailability of the drug is to improve drug efficacy or reduce toxicity due to the high dose of poorly available drugs. Athanassiou et al.’s study of several β-lactam antibiotics with cyclodextrins showed a positive correlation between the presence of cyclodextrin and increased minimal inhibitory concentration (MIC) of antibiotics [10]. Similar observations were made by our group in studies of βCD systems with meropenem [11] and tebipenem pivoxil [12], leading to the conclusion that antimicrobial efficacy tests should be conducted routinely for each antibiotic–CD system to assess change in the system’s pharmacodynamics compared to pure API.

The eligibility of certain kinds of cyclodextrin to achieve the desired stability of the system in solution is determined by the structure of CD and guest API [13]. The thermodynamic effect described by Gibbs free energy of complex formation, as well as by entailed stability constant Ks, can be estimated by structure-based modeling with in silico methods [11,12,14,15,16,17,18]. Application of theoretical approaches, considering a vast number of testable cyclodextrin derivatives, is an essential tool for streamlining experimental investigation.

The aim of this study was to evaluate the solubility and antimicrobial activity of CA in a system designed according to the guidance of validated in silico model. The subgoals include the curation of a literature dataset of experimental Gibbs free energies of API–CD system formation, validating the model based on the dataset, model application to screen CA–CD systems, preparation and determination of complex, evaluation changes in CA dissolution and antimicrobial profiles.

## 2. Materials and Methods

### 2.1. Study Design

**Figure 2:**
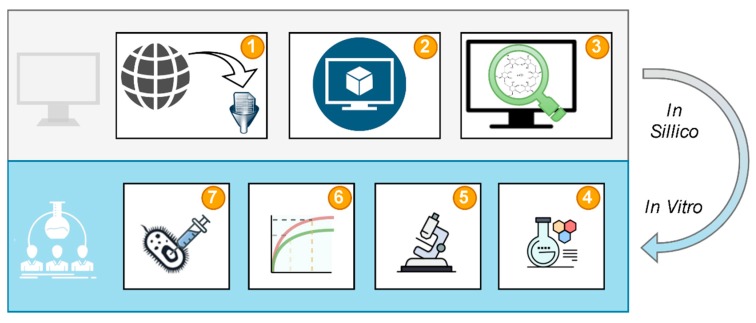
Study design: Data curation (**1**), model development (**2**), virtual screening (**3**), system preparation (**4**), characterization (**5**), dissolution tests (**6**), and antimicrobial efficacy tests (**7**).

The study design (Figure 2) shows the workflow followed to achieve the goals of the study. Data curation (Figure 2.1) provided molecular structures and experimental data for model validation (Figure 2.2). The validated model was used for virtual screening of cyclodextrins to select the most favored CD to form a system with CA (Figure 2.3). The most favored system by means of the predicted thermodynamic effect was prepared (Figure 2.4) and characterized using thermal, powder diffraction, and spectral methods (Figure 2.5). The change of CA solubility after the creation of the CD system was tested in dissolution studies (Figure 2.6). Antimicrobial efficacy of the system compared to pure CA was evaluated at the last stage of the study (Figure 2.7).

### 2.2. Data Curation

The dataset from a large consistent study [19] on Gibbs free energy of association (*d*G) of drug-like chemicals in systems with α- and β-cyclodextrins was curated according to workflow [20]. IUPAC names provided in the experimental study were resolved using ChemAxon Molconverter. In the subsequent step, the two-dimensional (2D) structures of molecules were generated and standardized using ChemAxon Standardizer. The three-dimensional (3D) structures of CDs for docking were based on crystal structures [21].

### 2.3. Model Validation

Docking of molecules from the dataset to structures of α- and β-cyclodextrins was done using AutoDock Vina [22]. The flexible ligands were docked to rigid CD on a grid spanning all of the CD. Computed poses were rescored using the Molecular Mechanics—Generalized Born Surface Area (MMGBSA) method implemented in the Schrodinger Prime 2019 package [23]. The predictive power of ranking the CD systems by affinity was assessed using the Pearson coefficient. No model parameters were tweaked, thus all data points were used to validate predictive power. The final docking pose was not affected by random initialization, and the procedure always converged to the same pose in the triplicated study.

### 2.4. Virtual Screening

Virtual screening ranked αCD, HP-αCD, βCD, HPβCD, and methyl-βCD (M-βCD) according to binding affinity predicted by the model. The same protocol as in model validation was applied to prepare CA and CDs for screening. CD derivatives were prepared in GaussView based on crystal structures of parent CDs, and reoptimized using Density Functional Theory with Becke, 3-parameter, Lee–Yang–Parr functional and 6-31G(d,p) basis set, implemented in Gaussian 09 [24].

### 2.5. System Preparation

Cefuroxime axetil (molar weight = 510 g/mol) was kindly provided by the Institute of Biotechnology and Antibiotics in Warsaw, Poland. HPβCD (molar weight = 1460 g/mol) was supplied by Sigma-Aldrich Chemie. The average molar substitution of CD was 0.8. Solvents used for system preparation were distilled water and analytical grade methanol. The systems were prepared with a co-precipitation method in 1:1 molar ratio. CA (765.0 mg) was dissolved in 10 mL of methanol and 2190.0 mg of HPβCD in 10 mL of water. The solutions were shaken in a rotary shaker at 60 rpm and 37 °C until complete evaporation of solvents. The residue sample was stored in a desiccator. Physical mixtures were prepared by gentle mixing equimolar amounts of CA and HPβCD in an agate mortar. The homogenized mixture was stored alongside co-precipitation samples in a desiccator.

### 2.6. System Determination

The thermal analysis of prepared samples was carried out with DSC apparatus TA Instruments DSC Q20, with the heating speed at 10 °C/min in the range 30–180 °C. The FT-IR spectra of systems, as well as pure substances constituting a given system along with their physical mixtures, were recorded with FT-IR Bruker IFS 66v/S with DTGS detector. The samples were put in KBr pills in a 1:100 ratio by applying 8 metric tonnes of pressure in a hydraulic press. The ATR spectra were obtained with ATR BRUKER VERTEX 70 with a DLaTGS detector directly on powder. The vibrational infrared spectra were measured between 400 and 4000 cm^−1^. The identification of the CA–HPβCD system was carried out via X-ray powder diffraction (PXRD). The XRD patterns of the samples were recorded on a Bruker AXS D2 Phaser diffractometer with Cu Kα radiation (λ = 1.54060 Å). The operating voltage and current were maintained at 30 kV and 10 mA, respectively. The samples were scanned from 5 to 45° 2θ. Selected higher-quality scans were made with a step size of 0.02°, with a counting rate of 2 s/step with the sample spinning. A 1 mm slit module was used during measurements. The acquired data were analyzed using the Origin Pro software [25].

### 2.7. Dissolution Studies

Apparent solubility profiles were determined using the USP standard paddle apparatus. The medium used for conducting the study was 500 mL water at 37 °C. The paddles’ rotation speed was 50 RPM. Into three jars, 5.0 mg of CA and an equal amount in respect to the mass of API of CA–HPβCD were added. At fixed time points, 5.0 mL of solution was taken from each jar and the medium in each jar was refilled with pure medium. Concentrations at each time point were measured using the UV spectrometer. The triplicated independent measurements were averaged and used to create a dissolution profile.

### 2.8. Microbiological Study

The bacterial strains analyzed with the MICs analysis included ATCC reference strains and clinical isolates from the Institute of Laboratory Medicine at Poznan, Poland. MIC was determined for each reference strain from the American Type Culture Collection and clinical isolates. MIC for cefuroxime axetil and CA with HPβCD was assayed using serial dilutions on the Mueller–Hinton liquid medium (Merck, Germany). In that experiment, microbial culture with standardized optical density was used. The applied method follows the standards of the National Committee for Clinical Laboratory Standards (NCCLS).

## 3. Results

### 3.1. In Silico Study

**Figure 3:**
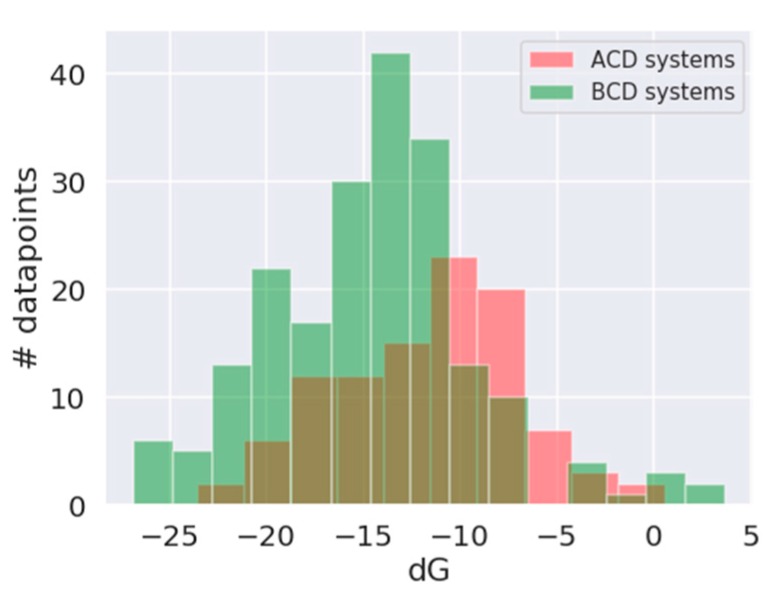
Distribution of *dG* in the dataset for API–αCD systems (red) and API–βCD systems (green).

All molecules in the original dataset were unique, resulting in a total of 102 data points of API–αCD and 202 datapoints of API–βCD systems. The distribution of data points followed normal distribution (Figure 3) with a mean *d*G value for αCD systems = −11.5 kJ/mol (K_s_ = 106.72 M^−1^) and a mean *d*G value for βCD systems = −14.52 kJ/mol (K_s_ = 350.41 M^−1^).

**Figure 4:**
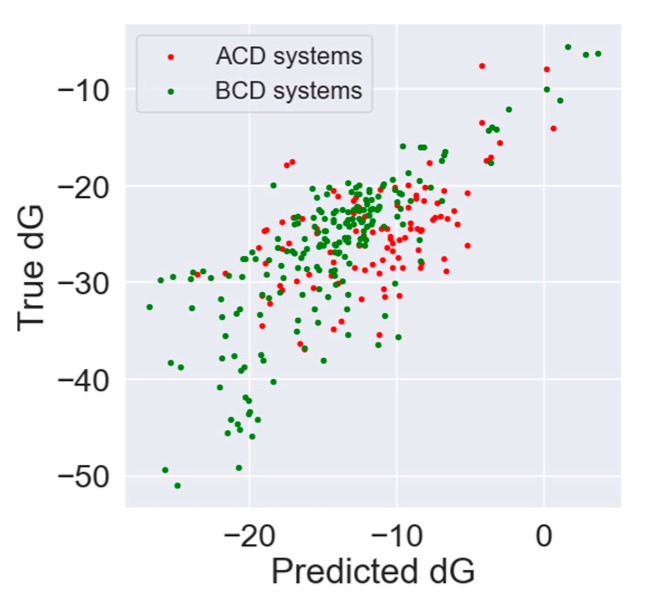
Autocorrelation plot of true and predicted *d*G for ACD systems (red) and BCD systems (green).

The predicted *d*G estimates were correlated with true *d*G values from the dataset to assess the model ability of correct ranking molecules by the thermodynamic effect. The Pearson correlation coefficient for αCD was 0.55, for βCD 0.77, and for the whole dataset, the model showed a correlation of 0.72. The autocorrelation plot of true and predicted values is presented in Figure 4.

Virtual screening with the model resulted in ranking scores for each of the tested CDs (Table 1). The HPβCD was predicted as the most favored one and was selected for experimental testing.

### 3.2. Experimental Study

#### 3.2.1. System Characterization

**Figure 5:**
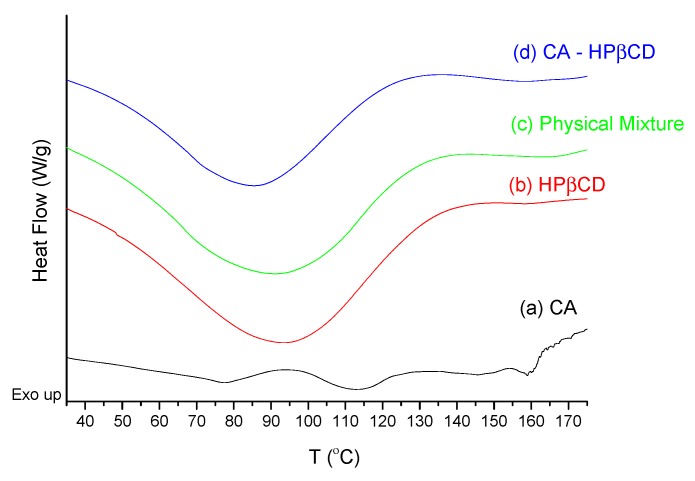
DSC thermograms of CA (**a**), HPβCD (**b**), their physical mixture (**c**), and system (**d**).

The CA–HPβCD system was characterized using thermal, spectral, and X-ray powder diffraction methods. Peaks at 77.3, 113.1, and 160 °C were observed on the DSC diagram of pure CA (Figure 5a). The peak at 77.3 °C is attributed to water loss from the sample, while the 113.1 °C and 160 °C peaks are melting points of two phases in CA sample. On the HPβCD thermogram (Figure 5b), the 92.4 °C peak attributed to water loss is observed. On a physical mixture thermogram (Figure 5c), one broad peak at 90.1 °C is observed. The thermogram of the physical mixture is a sum of the effects observed on the thermograms of pure constituents. On the thermogram of the CA–HPβCD system, one peak at 85.3 °C is observed with a low width relative to the physical mixture. The changes in peaks observed for physical mixture and the system may indicate the formation of a new phase solid [26]. Observed changes in thermograms were further investigated with spectral and XRPD techniques.

**Figure 6:**
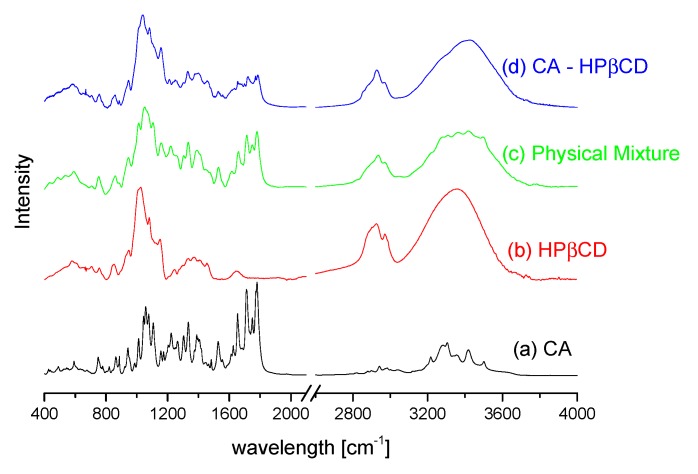
The FT-IR spectra of CA (**a**), HPβCD (**b**), their physical mixture (**c**), and prepared system (**d**).

The FT-IR spectra of CA, HPβCD, CA–HPβCD physical mixture, and CA–HPβCD system are presented (Figure 6). The analysis of differences between physical mixtures and systems allows elucidating possible binding domains present in the system. Differences in peaks intensities, as well as intensity quenching, are observed in the spectra. The most notable reduction in peak intensity occurs at 1710 cm^−1^ and 1778cm^−1^. The observed bands are related to vibration in carbonyl groups being present in axetil moiety (1710 cm^−1^) and β-lactam ring (1778 cm^−1^). The vibration of the carbonyl group is observed at 1652 cm^−1^ on the CA FT-IR spectrum, whereas in a CA–HPβCD sample spectrum, the peak is significantly reduced. Mentioned changes at highly polar chemical groups can be explained by the association of the polar fragments of HPβCD and CA. Changes at 1056 cm^−1^ are also observed, which may be related to the vibration of the methyl group in the axetil side chain. The peak is observed at 1051 cm^−1^ on the pure CA spectrum, 1052 cm^−1^ on the physical mixture spectrum, and is shifted to 1033 cm^−1^ on the spectrum of the system.

**Figure 7:**
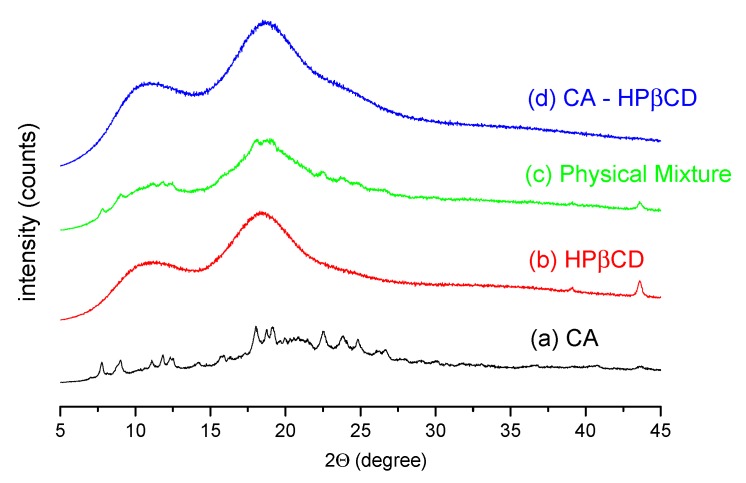
XRPD diffraction patterns of CA (**a**), HPβCD (**b**), physical mixture of CA–HPβCD (**c**), and prepared system (**d**).

The recorded powder diffraction pattern of the CA sample (Figure 7a) is a mixture of crystalline and amorphous forms, with sample crystallinity estimated at approximately 41% using the Diffrac.Eva software [27]. The diffraction peak positions are characteristic of the crystalline α form of CA [28,29]. The diffraction pattern of HPβCD (Figure 7b) indicates an amorphous form of cyclodextrin. The XRPD pattern of the physical mixture with a 1:1 molar ratio of CA and HPβCD (Figure 7c) is the sum of the diffractograms of pure compounds. The estimated percentage of sample crystallinity is 41%. The total loss of crystallinity of the sample occurs after the co-precipitation of CA and HPβCD in a 1:1 molar ratio from a water–methanol solvent (Figure 7d).

#### 3.2.2. Solubility Studies

**Figure 8:**
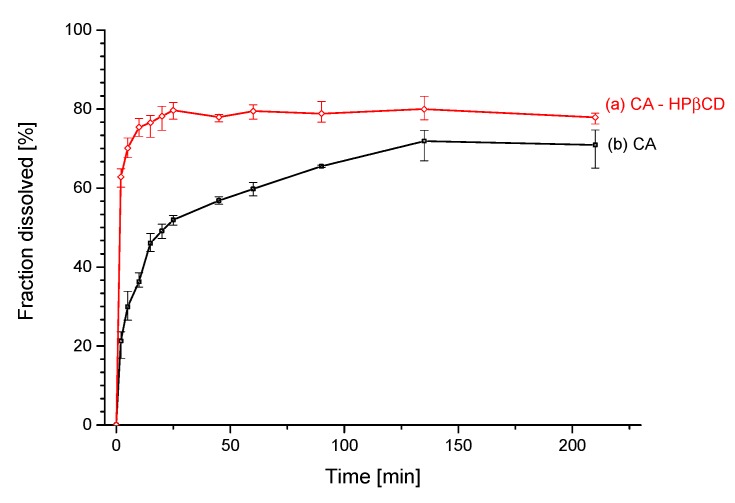
The apparent solubility profile of CA (**a**) and CA–HPβCD system (**b**).

The apparent solubility study indicates a steeper curve of changes of solubility for CA–HPβCD system than for pure CA (Figure 8). The formation of the system allowed to decrease the time of reaching a plateau of apparent solubility curve. After 2 min, the inclusion sample was dissolved in 63%, while the same quantity of pure CA was only dissolved in 21%. The plateau of the CA–HPβCD system was reached after 60 min at the level of 80% dissolved CA. The pure CA reached a plateau of 72% dissolved quantity after 165 min. The two apparent solubility profiles were compared using the relative difference factor f_1_ and relative similarity factor f_2_. The computed values were f_1_ = 49.48 and f_2_ = 28.74.

#### 3.2.3. Microbiological Study

MIC values were measured for selected bacterial species, including reference species as well as clinical isolates (Table 2). The presence of HPβCD had a significant effect on the length of bacterial survival. For CA, the lowest MIC was observed for *Klebsiella pneumoniae*—2 and 8 mg/L for reference species and clinical isolate, accordingly. Higher MIC values were observed for *Proteus mirabillis* and were equal 16 and 32 mg/L for reference species and clinical isolate, respectively. In parallel, the same tests were carried out for CA–HPβCD system. Application of CA–HPβCD decreased MIC values for all investigated species of Gram-negative bacteria. The highest degree of change in MIC values was observed for the clinical isolate *Klebsiella pneumoniae* (CA 8 mg/L → CA–HPβCD 2 mg/L) and the clinical isolate *Pseudomonas aeruginosa* (CA 32 mg/L → CA–HPβCD 8 mg/L).

## 4. Discussion

Designing a CA–CD system can be successfully streamlined using in silico methods. CA creates a stable system with HPβCD in solution, leading to significant changes in the dissolution profile and increase of antimicrobial activity for several Gram-negative bacteria species.

The validated model achieved a mean correlation with experimental data R > 0.7, which indicates an acceptable level of predictive performance. The in silico studies allowed to rank CA–CD systems by predicted affinities, thus to select the most favorable system. According to the published thermodynamic effect of complexation in the studies of CA–HPβCD (Ks = 382.7 M^−1^) [7] and CA–βCD (Ks = 339.7 M^−1^) [8], the predicted ranks 1 and 3, respectively, remain in agreement with experimental results. In the literature, good agreement of in silico predictions with experimental results was observed for Ibuprofen systems with β−, γ−, and HPβCD, where *d*G of binding was simulated with the MM–PBSA approach [30]. The study revealed the most energetically favored Ibuprofen–HPβCD system, the theoretical results were confirmed experimentally. The MM–GBSA method was also employed in the study of single CD in a system with Ibuprofen to model chiral discrimination of *S*-Ibuprofen and *R*-Ibuprofen by βCD [31], showing the possibility of ranking stereoisomers by binding affinity in CD systems. The results of our research and published studies confirm the usefulness of both MM–GBSA and MM–PBSA in the relative ranking of API–CD interactions and draw molecular mechanics simulations as a useful tool for streamlining the development of cyclodextrin systems.

The predicted most favored system was investigated experimentally. The solid-state system of CA–HPβCD was characterized with DSC, FT-IR, and XRPD techniques. According to the XRPD pattern of the system, the peaks from the CA crystalline fraction completely disappeared, which can be attributed to the interaction between CA and HPβCD in the co-precipitation process, leading to the formation of an amorphous system. A decrease in the peak on the DSC thermogram of the CA–HPβCD system by ~7 °C compared to those reported for CA and HPβCD was also observed for other systems prepared by the co-precipitation method [14]. It may be suggested that the preparation of the CA–HPβCD system using co-precipitation promotes dehydrogenation of cyclodextrin during the preparation of systems (changes at ~90 °C). In the FT-IR spectra, the most visible changes were observed in the intensities of bands from the CA carbonyl groups, which tends to interact with HPβCD hydroxyl groups [32]. The results of this study correlate with those obtained by Sapte, who also complexed CA with βCD using the spray-drying technique with or without the addition of L-arginine [8].

Changes in physicochemical properties of CA in the system with HPβCD were evaluated with profile of apparent solubility. The apparent solublity profile improved significantly, reaching a higher plateau on the solubility curve (80% after 60 min) compared to pure CA (72% after 165 min). Values of CA and CA–HPβCD apparent solubility profiles dissimilarity insignificance (f1) and similarity insignificance (f2) were f1 = 49.48 and f2 = 28.74. Compared to standard values, f1 > 15 indicates a significant difference between the two profiles, while f2 < 50 indicates that profiles are not significantly similar.

The ultimate goal of the study to increase the antimicrobial efficacy of CA was achieved for several species tested, with the most notable examples of a 4-fold increase in antimicrobial activity for clinical isolates of *Klebsiella pneumoniae* and *Pseudomonas aeruginosa.*

The increase in the bactericidal effect of the CA system can be the result of two factors. First, as a result of increased solubility, the ability to interact with proteins on the surface of the bacterial cell membrane (PBP) increases as well. Second, due to the stabilizing effect of cyclodextrins on the chemical stability of β-lactam antibiotics [33], the effect of hydrolysis leading to breaking β-lactam bond may be reduced. Moreover, the impact of cyclodextrin on bacterial cells should be considered. In the literature, information can be found about several compounds (including βCD), which inhibit the α-hemolysin cytotoxicity of *Staphylococcus* *aureus*. An increase in antibacterial activity was also observed in the cases of cefdinir–βCD and meropenem–βCD complexes [11,34]. Taking into account a significant decrease of *Klebsiella pneumoniae* and *Pseudomonas aeruginosa* MICs for the CA–HPβCD system, two pathways leading to increased efficacy may be suggested. Firstly, blocking porin channels may be contributed to inhibiting the efflux effect in bacteria by HPβCD [12]. Limitations of efflux transport in the case of the derivatives of cyclodextrin were observed by the authors as early as the stage of the permeability studies. Secondly, HPβCD can interact with zinc ions, which are active centers in β-lactamases. As a consequence, resistance is not induced among selected bacterial strains.

## 5. Conclusions

A dataset of API–CD values of *d*G of formation was curated and used for successful validation of the model. The model showed acceptable predictive performance, thus it was used for virtual screening of CA–CD systems. The most thermodynamically favored system, CA–HPβCD, was prepared and determined with thermal, spectral, and XRPD analytical methods. Dissolution and microbiological studies revealed significant improvement in both solubility and antimicrobial efficacy of CA in a system with HPβCD.

## Figures and Tables

**Table 1:** Virtual screening results.

	Absolute Value [kJ/mol]	Relative Value [%]	Rank Prediction
HPβCD	−57.84	100.00	1
RMβCD	−56.64	97.91	2
βCD	−51.21	88.53	3
αCD	−50.55	87.38	4
HPαCD	−49.24	85.13	5

**Table 2:** The minimal inhibitory concentration (MIC) values measured for CA and CA–HPβCD complex on different species of Gram-negative bacteria.

Microorganism	MIC (mg/L)	
	CA	CA–HPβCD
*Proteus mirabilis* ATCC 12453	16	8
*Proteus mirabilis* clinical isolates	32	16
*Klebsiella pneumoniae* ATCC 31488	2	1
*Klebsiella pneumoniae* clinical isolates	8	2
*Enterobacter aerogenes* ATCC 13048	64	32
*Enterobacter aerogenes* clinical isolates	128	64
*Enterococcus faecalis* ATTC 29212	64	64
*Enterococcus faecalis* clinical isolates	128	128
*Escherichia coli* ATCC 25922	64	32
*Escherichia coli* clinical isolates	64	32
*Staphylococcus aureus* ATCC 25923	32	32
*Staphylococcus aureus* clinical isolates	32	32
*Acinetobacter baumanii* ATCC 19606	32	16
*Acinetobacter baumanii* clinical isolates	64	32
*Pseudomonas aeruginosa* ATCC 27853	16	8
*Pseudomonas aeruginosa* clinical isolates	32	8
